# Sesamin inhibits RANKL‐induced osteoclastogenesis and attenuates LPS‐induced osteolysis via suppression of ERK and NF‐κB signalling pathways

**DOI:** 10.1111/jcmm.18056

**Published:** 2023-11-21

**Authors:** Xiaolong Yu, Jiawei Hu, Xinming Yang, Qiang Xu, Hangjun Chen, Ping Zhan, Bin Zhang

**Affiliations:** ^1^ Orthopedics Department The First Affiliated Hospital of Nanchang University, Artificial Joints Engineering and Technology Research Center Nanchang China

**Keywords:** ERK, NF‐κB, osteoclast, Osteolysis, Sesamin

## Abstract

Infection by bacterial products in the implant and endotoxin introduced by wear particles activate immune cells, enhance pro‐inflammatory cytokines production, and ultimately promote osteoclast recruitment and activity. These factors are known to play an important role in osteolysis as well as potential targets for the treatment of osteolysis. Sesamin has been shown to have a variety of biological functions, such as inhibiting inflammation, anti‐tumour and involvement in the regulation of fatty acid and cholesterol metabolism. However, the therapeutic effect of sesamin on osteolysis and its mechanism remain unclear. Present studies shown that in the condition of in vitro, sesamin could inhibit osteoclastogenesis and bone resorption, as well as suppressing the expression of osteoclast‐specific genes. Further studies on the mechanism suggest that the effect of sesamin on human osteoclasts was mediated by blocking the ERK and NF‐κB signalling pathways. Besides, sesamin was found to be effective in treating LPS‐induced osteolysis by decreasing the production of pro‐inflammatory cytokines and inhibiting osteoclastogenesis in vivo. Sesamin was non‐toxic to heart, liver, kidney, lung and spleen. Therefore, sesamin is a promising phytochemical agent for the therapy of osteolysis‐related diseases caused by inflammation and excessive osteoclast activation.

## 1 INTRODUCTION

Osteolytic diseases, including osteoporosis, rheumatoid arthritis, Paget's disease, bone metastatic cancer, periprosthetic joint infection and periprosthetic loosening, are characterized by bone loss that could result in pathological fractures.^1^ Among these diseases, osteolysis in periprosthetic joint infection and periprosthetic loosening is activated by increased activation of osteoclastic bone resorption, which is mainly caused by bacterial infection after implant installation and by endotoxins introduced by wear particles.^2^, ^3^ Lipopolysaccharide (LPS), is a major component of the cell wall of gram‐negative bacteria, also called as endotoxin synonymously that promotes osteoclast formation and enhances inflammatory osteolysis.^4^ LPS‐induced osteolysis promotes the secretion of several bioactive pro‐inflammatory cytokines, such as tumour necrosis factor alpha (TNF‐α), interleukin 6 (IL‐6) and interleukin 1β (IL‐1β), which promote osteoclast recruitment and activity, ultimately leading to osteolysis.^5^, ^6^ Therefore, inhibition of pro‐inflammatory cytokines secretion and osteoclast activation is an effective osteoprotective therapy for the treatment of LPS‐induced osteolysis.

Studies have shown that receptor activator of nuclear factor‐κB ligand (RANKL) and macrophage colony‐stimulating factor (M‐CSF) are two key cytokines for osteoclast differentiation and maturation.^7^ LPS generated from wear particles of artificial joints can produce pro‐inflammatory cytokines and induce osteoblasts to increase RANKL expression.^8^, ^9^ Following RANKL binding to RANK, TRAF6 is recruited to the cytoplasmic domain, which leads to the activation of mitogen‐activated protein kinases (MAPKs) and the transcription factors nuclear factor‐κB (NF‐κB).^10^ In the NF‐κB signalling pathway, many extracellular stimuli, including LPS, cytokines and viruses, activate NF‐κB by inducing phosphorylation of the kinase IKK resulting in degradation ofIκBα, which releases active, dimeric NF‐κB.^11^ After being activated, NF‐κB is translocated into the nucleus and binds to specific DNA fragments, resulting in osteoclast‐specific gene transcription which leads to osteoclast differentiation and maturation.^12^, ^13^ In addition, MAPK is a group of serine/threonine protein kinases in cells that includes three pathways, ERK, P38 and JNK.^14^ These above proteins are activated by phosphorylation to regulate gene transcription and initiate the regulation of osteoclast precursor cell differentiation and bone resorption functions, which in turn are closely associated with the progression of osteolytic diseases.^15^ Therefore, studying the mechanism of osteoclast formation and bone resorption function and inhibiting its progression may bring benefits to the prevention and treatment of osteolytic diseases.

In recent years, plant‐derived natural products are a treasure trove for exploring new therapeutic approaches for clinical diseases due to their easy availability, low price, low side effects and multi‐targeted effects.^16^ Sesame (*Sesamum indicum* L.) is a food that could potentially be considered a medicine and belongs to the legume family. Sesame seeds are rich in phytochemicals called lignans, which are methylenedioxyphenyl compounds. The medicinal properties in sesame oil are mainly due to 0.5%–1.1% sesamin, 0.2%–0.6% sesamolin and traces of sesamol.^17^ Sesamin has been proven to have a variety of biological functions, such as inhibition of inflammation, anti‐tumour and participating in regulation of fatty acid and cholesterol metabolism.^18^, ^19^ However, to our knowledge, whether sesamin can attenuate LPS‐induced osteolysis by inhibiting osteoclast differentiation has not been revealed.

Therefore, we performed the following experiments presented in this study. (i) investigate whether sesamin inhibits RANKL‐induced osteoclastogenesis, and (ii) to reveal the potential mechanisms underlying the effects of sesamin on osteoclast formation and function, and (iii) to elucidate the potential therapeutic role of sesamin in inhibiting LPS‐induced osteolysis. The results presented here suggest a promising natural products that may have the potential to prevent osteolysis diseases and provide a theoretical basis for the clinical development of novel drugs for the treatment of bone loss related diseases.

## 2 MATERIALS AND METHODS

### 2.1 Chemicals, reagents and antibodies

**FIGURE 1:**
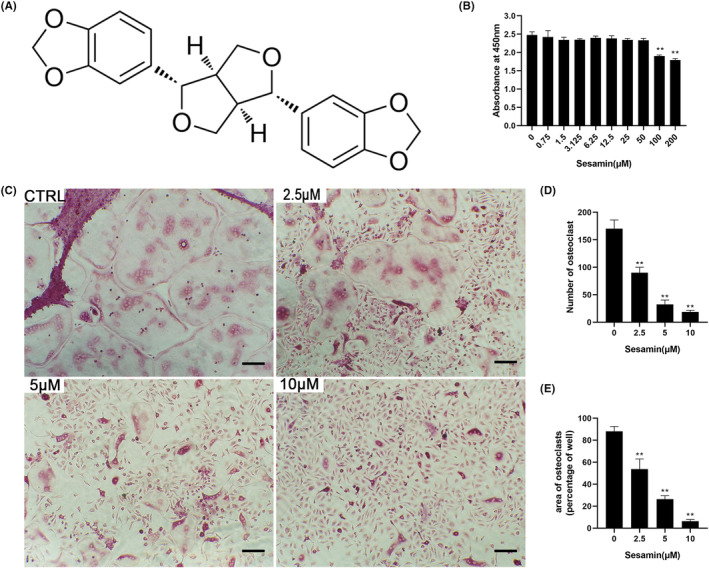
Sesamin inhibited RANKL‐induced osteoclastogenesis without cytotoxic effects. (A) The structure of sesamin. (B) BMMs stimulated by M‐CSF (30 ng/mL) with different concentrations of sesamin for 48 h. Cell viability was measured using the CCK‐8 assay. (C) BMMs stimulated by M‐CSF (30 ng/mL) and RANKL (50 ng/mL) with or without different concentrations of sesamin for 5–7 days, then the cells were stained for TRAP. Scale bar = 200 μm. (D, E) TRAP‐positive multinuclear cells numbers and area percentage were counted. Data are presented as the mean ± SD (*p < 0.05 and **p < 0.01, vs. control group).

(C20H18O6, purity ≥ 98%) was purchased from Chengdu must Biological Technology Co., Ltd. (Chengdu, China). Figure 1A showed the chemical structure of sesamin. RAW264.7 cells were obtained from American Type Culture Collection (ATCC, Rockville, MD, United States). FBS (Fetal bovine serum), α‐MEM (alpha modification of Eagle medium) and 1% penicillin/streptomycin were purchased from Gibco‐BRL (Sydney, Australia). Recombinant murine M‐CSF and recombinant murine RANKL was purchased from R&D Systems (Minneapolis, MN, United States). Cell Counting Kit‐8 (CCK‐8) was purchased from Dojindo Molecular Technology (Kumamoto, Japan) and TRAP (Tartaric acid resistant acid phosphatase) enzyme activity kit was purchased from Sigma‐Aldrich (Minneapolis, MN, United States). Primary antibodies against IκBα, ERK, phospho‐ERK, JNK, phospho‐JNK, p38, phospho‐p38, p65, phospho‐p65, NFATC1, C‐FOS, Cathepsin K and GAPDH was obtained from Cell Signaling Technology (Danvers, MA, United States). Alendronate sodiuma, tert‐Butylhydroquinone (tBHQ) and Betulinic acid (BetA) were purchased from Medchemexpress (shanghai, China).

### 2.2 Cell viability assay

Based on manufactur's instructions, the effects of sesamin on BMMs (Bone marrow‐derived macrophages) viability was depended the CCK‐8 assay. Briefly, BMMs were seeded in triplicates in 96‐plates well at a density of 8 × 10^3^ cells for each well and cultured in complete medium supplement of α‐MEM containing 30 ng/mL M‐CSF with increasing concentrations of sesamin, tBHQ, and BetA. At the end of the experimental procedure, each well was added with a buffer solution at 10 μL CCK‐8. Absorbance measurement was done through an ELX800 microplate reader (Bio‐Tek Instruments Inc.; Winooski, VT, United States) at a wavelength of 450 nm. Cell viability was expressed as proportion‐relate for formula controlling (experimental group OD ‐ zeroing OD) versus (control group OD ‐zeroing OD).

### 2.3 Cell Culture and Osteoclast Differentiation Assay

BMMs was isolated lower bilateral lower limb femurs and tibias of 4 ~ 6 weeks C57BL/6 mice (Hunan SJA Laboratory Animal Co., Ltd, Hunan China). After then, BMMs were cultured in α‐MEM complete medium containing 10% fetal bovine serum, 1% penicillin/streptomycin and 30 ng/mL M‐CSF in T75 culture flasks for 24 hours. The suspension of cells was removed and the adherent cells continued to be cultured in the above medium until the cells were completely fused. Next, BMMs with were seeded in triplicate at density of 8 × 10^3^ cells/well into 96‐well plate and cultured with complete α‐MEM medium containing 30 ng/mL M‐CSF and 50 ng/mL RANKL for 5–6 days with various concentrations of sesamin (0, 2.5, 5, 10 μM). The medium was renewed every other day until mature osteoclast were formed within microscope. Using the manufacturer's recommended protocol, cells were washed using phosphate buffered saline and fixed with 4% paraformaldehyde and then stained for TRAP activity. TRAP‐stained cells with three or more than three nuclei were scored as osteoclasts.

### 2.4 F‐Actin ring staining

We seeded BMMs onto Sterile glass coverslips into 24‐well plates at a density of 4 × 10^4^ cells/well. The cells were treated with different concentrations of sesamin (0, 5 and 10 μM), and then cells were induced every other day with complete α‐MEM supplemented with 30 ng/mL M‐CSF and 50 ng/mL RANKL. After osteoclast formation, cells were fixed with 4% paraformaldehyde for 20 min at room temperature. It was then permeabilised with 0.1% Triton X‐100 (Sigma‐Aldrich, Minneapolis, MN, United States) for 5 min and washed three times with phosphate‐buffered saline. Cell nuclei were stained with DAPI, and then the stained cells were observed using an LSM5 (Carl Zeiss, Oberkochen, Germany) confocal microscope to reveal fluorescent signals and capture representative images. Finally, the number of nuclei and area of osteoclasts were analysed in conjunction with Zeiss ZEN software.

### 2.5 Bone resorption assay

We seeded BMMs onto bone slice of sterile bovine into 96‐well plates at a density of 1 × 10^4^ cells/well. The cells were treated with different concentrations of sesamin (0, 5 and 10 μM), and then cells were induced every other day with complete α‐MEM supplemented with 30 ng/mL M‐CSF and 50 ng/mL RANKL. Once mature osteoclasts appeared in the control group, treatment of the cells was continued for 48 h. Osteoclasts attached to the bovine bone slices were subsequently removed using an ultrasonic oscillator. Then, bone resorption pits were observed by scanning electron microscopy (FEI Quanta 250, Hillsboro, USA) and the area of bone resorption was quantified using ImageJ software (Bethesda, MD, USA).

### 2.6 RNA extraction and quantitative PCR assay

We proceeded in two groups, one in which BMMs were seeded in six‐well plates at a density of 1 × 10^5^ cells/well and cultured them in complete α‐MEM supplemented with 30 ng/mL M‐CSF and 50 ng/mL RANKL. Cell culturing within 0, 5 and 10 uM sesamin for 5–7 days till formation of the mature osteoclast. The other set consisted of BMMs seeded at a density of 1 × 10^5^ cells/well in six‐well plates and cultured them in complete α‐MEM supplemented with 30 ng/mL M‐CSF. Cell culture was divided into four columns without Alendronate sodium and RANKL, and without sesamin; 50 ng/mL RANKL; 5 μM sesamin + 50 ng/mL RANKL; 5 μM sesamin + 50 ng/mL RANKL; and 5 μM Alendronate sodium+50 ng/mL RANKL, respectively, and was cultured for 5–7 days until mature osteoclasts were formed. Total RNA was extracted by TRIzol reagent (Thermo Fisher Scientific) and cDNA was synthesized from 1 mg of total RNA using reverse transcriptase (TaKaRa, Otsu, Japan). The cDNA samples were analysed for genes of interest and reference genes. Furthermore, SYBR® Premix Ex Taq™ kit (TaKaRa, Otsu, Japan) and ABI StepOnePlus System (Applied Biosystems, Inc., Foster City, CA, United States) was implemented to conduct qPCR in detection system. QPCR was performed for 40 cycles, with denaturation at 95°C for 5 s, amplification at 60°C for 10 s, and GAPDH was used as a standardized osteoclast‐specific gene. The primer sequences of TRAP, Cathepsin K, nuclear factor of activated T‐cells, cytoplasmic 1 (NFATC1), calcitonin receptor (CTR), V‐ATPased2, dendritic cell‐specific transmembrane protein (DC‐STAMP), V‐ATPase a3, c‐fos, and GAPDH were as follows: TRAP Forward, 5′‐TCATGGGTGGTGCTGCT‐3′ and Reverse, 5′‐GCCCACAGCCACAAATCT‐3′; Cathepsin K Forward, 5′‐CTTCCAATACGTGCAGCAGA‐3′ and Reverse, 5′‐TCTTCAGGGCTTTCTCGTTC‐3′; NFATc1 Forward, 5′‐GAGTACACCTTCCAGCACCTT‐3′ and Reverse, 5′‐TATGATGTCGGGGAAAGAGA‐3′; CTR Forward, 5′‐TGCAGACAACTCTTGGTTGG‐3′ and Reverse, 5′‐TCGGTTTCTTCTCCTCTGGA‐3′; V‐ATPased2 Forward, 5′‐AAGCCTTTGTTTGACGCTGT‐3′ and Reverse, 5′ ‐TTCGATGCCTCTGTGAGATG‐3′; DC‐STAMP Forward, 5′‐AAAACCCTTGGGCTGTTCTT‐3′ and Reverse, 5′‐AATCATGGACGACTCCTTGG‐3′; V‐ATPase a3 Forward 5′‐GCCTCAGGGGAAGGCCAGATCG‐3′, and Reverse, 5′‐GGCCACCTCTTCACTCCGGAA‐3′; c‐fos Forward, 5′‐CCAGTCAAGAGCATCAGCAA‐3′ and Reverse, 5′ ‐AAGTAGTGCAGCCCGGAGTA‐3′; GAPDH Forward, 5′‐ACCCAGAAGACTGTGGATGG‐3′ and Reverse, 5′‐CACATTGGGGGTAGGAACAC‐3′.

### 2.7 Western blot

RAW264.7 cells were evenly seeded in a six‐well plates at a density of 5 × 10^5^ cells/well. When the cells grew to more than 80%, they were divided into eight groups. The first group was divided into four columns without sesamin and RANKL; 50 ng/mL RANKL; 5 μM sesamin + 50 ng/mL RANKL; and 10 μM sesamin + 50 ng/mL RANKL, respectively. The second group was divided into 12 columns, pretreated with 5 μM sesamin and without sesamin, and then stimulated with 50 ng/mL RANKL for 0, 5, 10, 20, 30, and 60 min, respectively. The third group was divided into five columns without tBHQ and RANKL; 50 ng/mL RANKL; 6.25 μM tBHQ + 50 ng/mL RANKL; 12.5 μM tBHQ + 50 ng/mL RANKL; and 25μM tBHQ + 50 ng/mL RANKL. The fourth group was divided into five columns without tBHQ and RANKL, and without sesamin; 50 ng/mL RANKL; 5 μM sesamin + 50 ng/mL RANKL; and 5 μM sesamin + 50 ng/mL RANKL + 12.5 μM tBHQ. The fifth group was divided into five columns without BetA and RANKL;50 ng/mL RANKL; 6.25 μM BetA + 50 ng/mL RANKL;12.5 μM BetA + 50 ng/mL RANKL; and 25 μM BetA + 50 ng/mL RANKL. The sixth group was divided into five columns without BetA and RANKL, and without sesamin; 50 ng/mL RANKL; 5 μM sesamin + 50 ng/mL RANKL; and 5 μM sesamin + 50 ng/mL RANKL + 25 μM BetA. The seventh group was divided into four columns without Alendronate sodium and RANKL, and without sesamin; 50 ng/mL RANKL; 5 μM sesamin + 50 ng/mL RANKL; 5 μM sesamin + 50 ng/mL RANKL; and 5 μM Alendronate sodium + 50 ng/mL RANKL, respectively. Subsequently, 2.5 μL of protease inhibitor, 2.5 μL of protein phosphatase inhibitor and 245 μLof RIPA lysate were added to each well, and the cell lysate was collected by ice lysis for 30 min. After centrifugation at 4°C, 12,000 g, for 10 min, the cell precipitate debris was removed and the protein concentration was determined by the BCA assay. The protein samples were subjected to gel electrophoresis and transferred to PVDF membrane. Moreover, the membranes were blocked with 5% (w/v) skim milk or bovine serum albumin for 1 h at room temperature. Subsequently, after co‐incubation with primary antibody overnight at 4°C. The membranes were washed three times with TBST for 10 min each, and then co‐incubated with the secondary antibody for approximately 1 h at room temperature. Secondary antibodies were washed and developed with chemiluminescence Western Blot detection kit (BestBio, Shanghai, China). Protein bands Exposure were performed using Odyssey Infrared Imaging System (Li‐COR Biosciences, Lincoln, NE, United States) to detect and quantify antibody reactivity. Finally, analysis of protein bands density were performed using ImageJ software.

### 2.8 Molecular docking

ERK (PDB ID: 2Y9Q) and p65 (PDB ID: 1LE9).^20^, ^21^ were selected for molecular docking targeting with sesamin. Firstly, we downloaded PDB format files of ERK and p65 receptors from the PDB database (https://www.rcsb.org/) and 3D conformational format files of sesamin ligands from the pubchem database (https://pubchem.ncbi.nlm.nih.gov/). Secondly, simulated protein modifications and receptor‐ligand docking analysis were then performed using AutoDockTools (version 1.5.6), followed by determination of the minimum binding energy of the ligand‐receptor binding pocket. Finally, docking images were visualized using PyMOL (version 2.40).

### 2.9 LPS‐induced mouse model of calvarial osteolysis

We established a mice model of LPS‐induced calvarial osteolysis to study the effect of sesamin in preventing and treating osteolysis. All experiments were conducted in accordance with the guidelines of the Animal Ethics Committee of the First Affiliated Hospital of Nanchang University. In short, 18 healthy 8‐week‐old female C57BL/six mice (Hunan SJA Laboratory Animal Co., Ltd, Hunan China) were randomly divided into three groups: Sham group (injection of PBS, *n* = 6), LPS group (injection of 5 mg/kg LPS, *n* = 6) and LPS + Sesamin group (injection of 5 mg/kg LPS and 10 mg/kg sesamin, *n* = 6). Under light anaesthesia, the reagents prepared for the corresponding three groups were injected subcutaneously at mice's daily calvaria sagittal midline suture for 14 days. Subsequently, calvaria samples were taken and 4% paraformaldehyde was fixed for 48 h for analysing Micro‐CT and histology.

### 2.10 Micro‐CT scanning

High‐resolution micro‐CT (Skyscan1172) was used to analyse of fixed calvaria samples. The scanning protocol was set at a 8.3 μm equidistant definition with 80 kV and 80 mA X‐ray energy settings. Following reconstruction, a square region of interest around the midline suture was selected for further qualitative and quantitative analysis. The bone volume versus tissue volume (BV/TV) and percentage of total porosity within regions of interest were analysed for each sample.

### 2.11 Histological and histomorphometric

The calvaria samples were decalcified with 10% EDTA within 3 weeks and thus embedded in paraffin. Bone tissue sections were selected for further analysis by haematoxylin and eosin staining and TRAP staining under the microscopic observation. TRAP‐positive multinucleated osteoclasts were quantified in all cranial samples using ImageJ software. Finally, to further verify the toxic effects of sesamin in vivo, the heart, liver, kidney, lung and spleen of perfused mice were taken and analysed by HE staining.

### 2.12 Enzyme‐linked immunosorbent assay (ELISA)

According to the manufacturer's requirements, blood was collected from mice and then serum was isolated, followed by determination of pro‐inflammatory cytokines. In briefly, ELISA kits was performed to measure the concentrations of TNF‐α, IL‐6 and IL‐1β. Next, horseradish peroxidase (HRP)‐coupled affinity proteins were added to each microplate well and incubated. After adding TMB substrate solution, the enzyme‐substrate reaction was terminated by adding sulfuric acid solution, and the colour change was measured spectrophotometrically a wavelength of 450 ± 10 nm. Pro‐inflammatory cytokines expression were then determined for each sample by comparing the OD values of the samples to a standard curve.

### 2.13 Statistical analysis

All data are presented as the mean ± SD (standard deviation). All experiments were performed at least three times independently. Statistical differences were based on Student's *t*‐test or analysis of variance (anova) analysis with GraphPad Prism software version 8.0 (GraphPad Software, San Diego, CA, USA). *p*‐values < 0.05 were considered statistically significant.

## 3 RESULTS

### 3.1 Sesamin inhibits RANKL‐induced osteoclast formation

Firstly, we examined the effect of sesamin on BMMs viability by the CCK‐8 assay. Our data revealed that the dose of sesamin <100 μM had no cytotoxic effect on BMMs (Figure 1B). We next studied the effect of sesamin on RANKL‐induced osteoclast formation. BMMs were treated with 30 ng/mL M‐CSF and 50 ng/mL RANKL at different concentrations of sesamin (0, 2.5, 5 and 10 μM) and cultured for 5–7 days. TRAP staining showed a gradually decreasing number and area of mature multinucleated osteoclasts with increasing sesamin concentration, which indicated that sesamin inhibited osteoclast formation dose‐dependently (Figure 1C–E).

### 3.2 Sesamin inhibits osteoclast function in vitro

**FIGURE 2:**
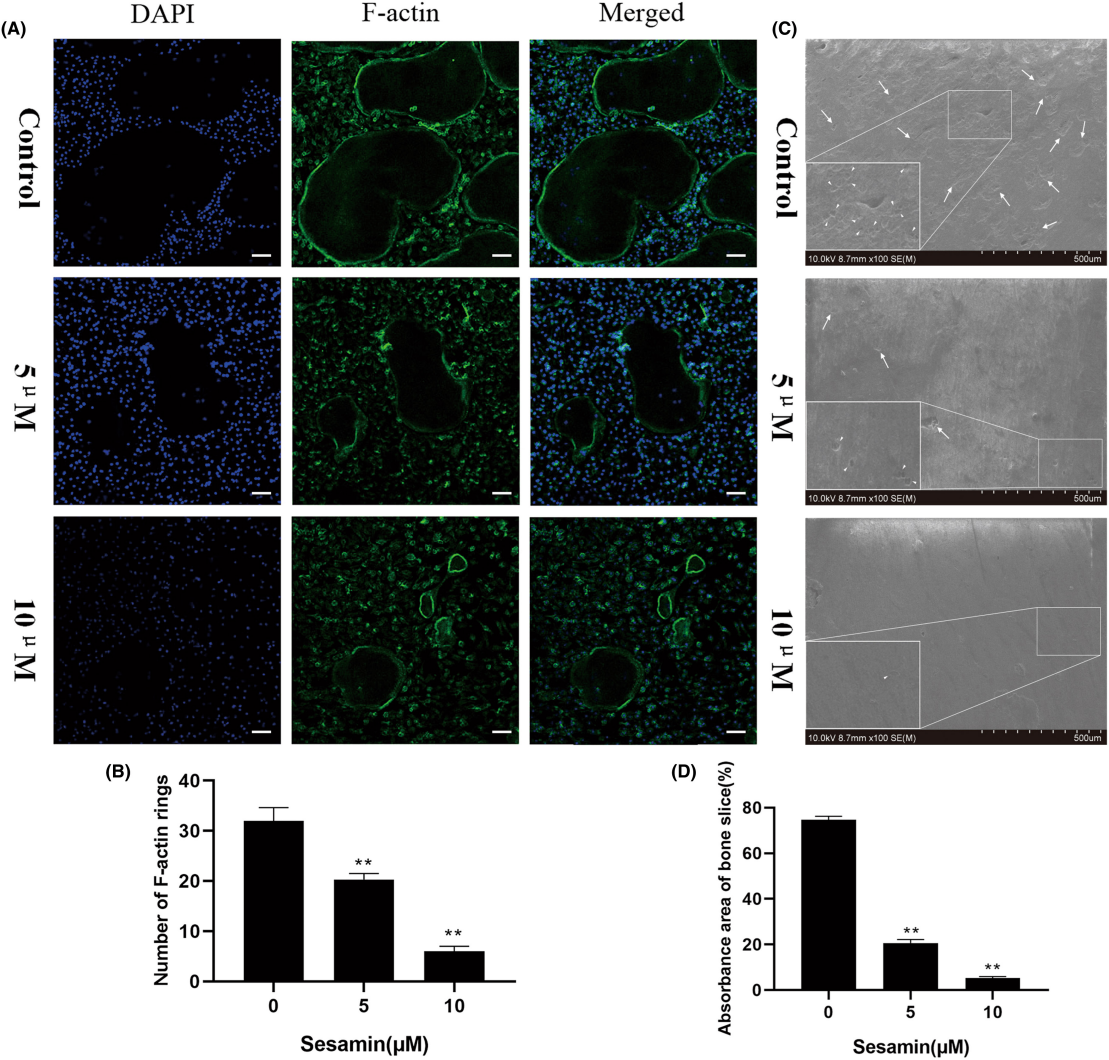
Sesamin inhibited RANKL‐induced F‐Actin rings formation and bone resorption. (A) F‐Actin rings confocal microscopy results. Scale bar = 200 μm. (B) Quantification of F‐Actin rings. (C) Scanning electron microscope images of bone resorption pits. Scale bar = 500 μm. (D) Percentage of resorption area. Data are presented as the mean ± SD (*p < 0.05 and **p < 0.01, vs. control group, The results shown in the figure are obtained by using BMMs).

F‐actin rings play a crucial function in osteoclast‐mediated bone resorption. Therefore, We investigated the effect of sesamin on the formation of F‐actin rings in osteoclastogenesis induced by RANKL in BMMs and quantified F‐actin rings using an immunofluorescence assay. The results showed that F‐actin rings were well polarized along with osteoclastogenesis induced by RANKL. Interestingly, sesamin treatment greatly reduced the number and area of F‐actin rings, which further demonstrated the ability of sesamin to inhibit osteoclast function (Figure 2A,B). Next, we further studied the effect of sesamin on osteoclast‐mediated bone resorption. Consistent with the F‐actin ring assay results, bone resorption pit formation was clearly observed on the surface of the control group treated with RANKL, whereas sesamin treatment reduced the area of bone resorption pits. The quantitative results showed that compared with the control group, the area of bone resorption pits were reduced by approximately 50% and 70% after 5 and 10 μM sesamin treatment, respectively (Figure 2C,D).

### 3.3 Sesamin suppressed RANKL‐induced osteoclast‐specific gene and protein expression

**FIGURE 3:**
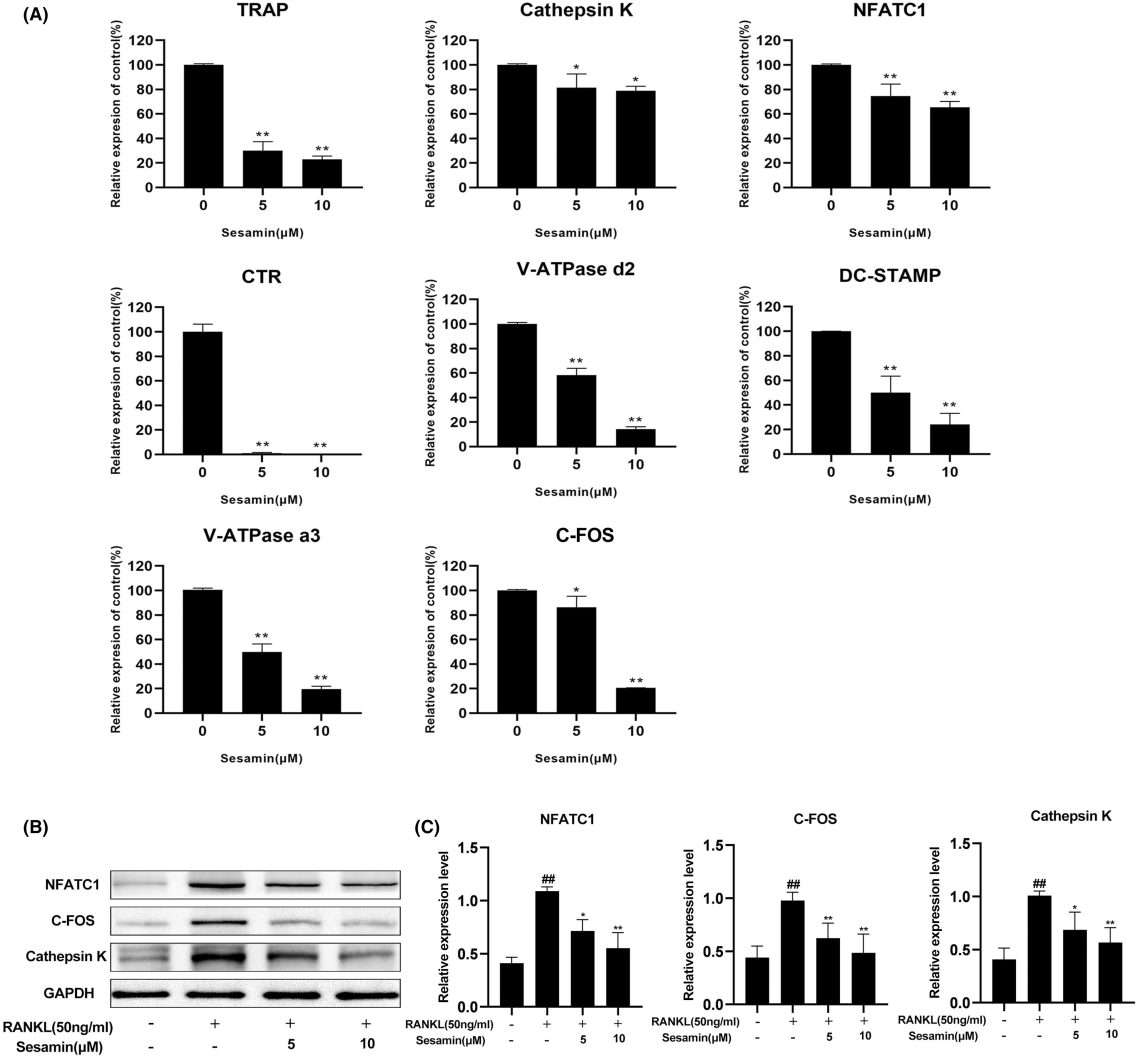
Sesamin suppressed RANKL‐induced osteoclast‐specific gene and protein expression. (A) Sesamin treatment inhibited the expression of TRAP, Cathepsin K, NFATc1, CTR, V‐ATPased2, DC‐STAMP, V‐ATPase a3, and C‐FOS genes relative to the RANKL‐treated control group in BMMs. (B) The protein expression levels of NFATC1, C‐FOS and Cathepsin K was significantly inhibited after sesamin treatment in RAW264.7.(C) Quantification of western blot analysis. Data are presented as the mean ± SD (^##^ *p* < 0.01 vs. the control group and *p < 0.05 and **p < 0.01 vs. the RANKL alone treatment group).

To further investigate the potential mechanism of sesamin on RANKL‐induced osteoclast formation, we used RT‐qPCR to detect the mRNA expression levels of RANKL‐induced osteoclast‐specific genes. The results showed that sesamin treatment inhibited the expression of TRAP, Cathepsin K, NFATc1, CTR, V‐ATPased2, DC‐STAMP, V‐ATPase a3 and C‐FOS genes relative to the RANKL‐treated control group. These results revealed that sesamin inhibited osteoclastogenesis by inhibiting the expression of osteoclast‐specific genes in vitro (Figure 3A). At the same time, we performed western blotting to measure the effect of sesamin on the expression levels of osteoclast‐specific proteins. The results revealed that the protein expression levels of NFATC1, C‐FOS and Cathepsin K was significantly inhibited after sesamin treatment (Figure 3B,C).

### 3.4 Sesamin and alendronate sodium inhibited RANKL‐induced osteoclast‐specific gene and protein expression

**FIGURE 4:**
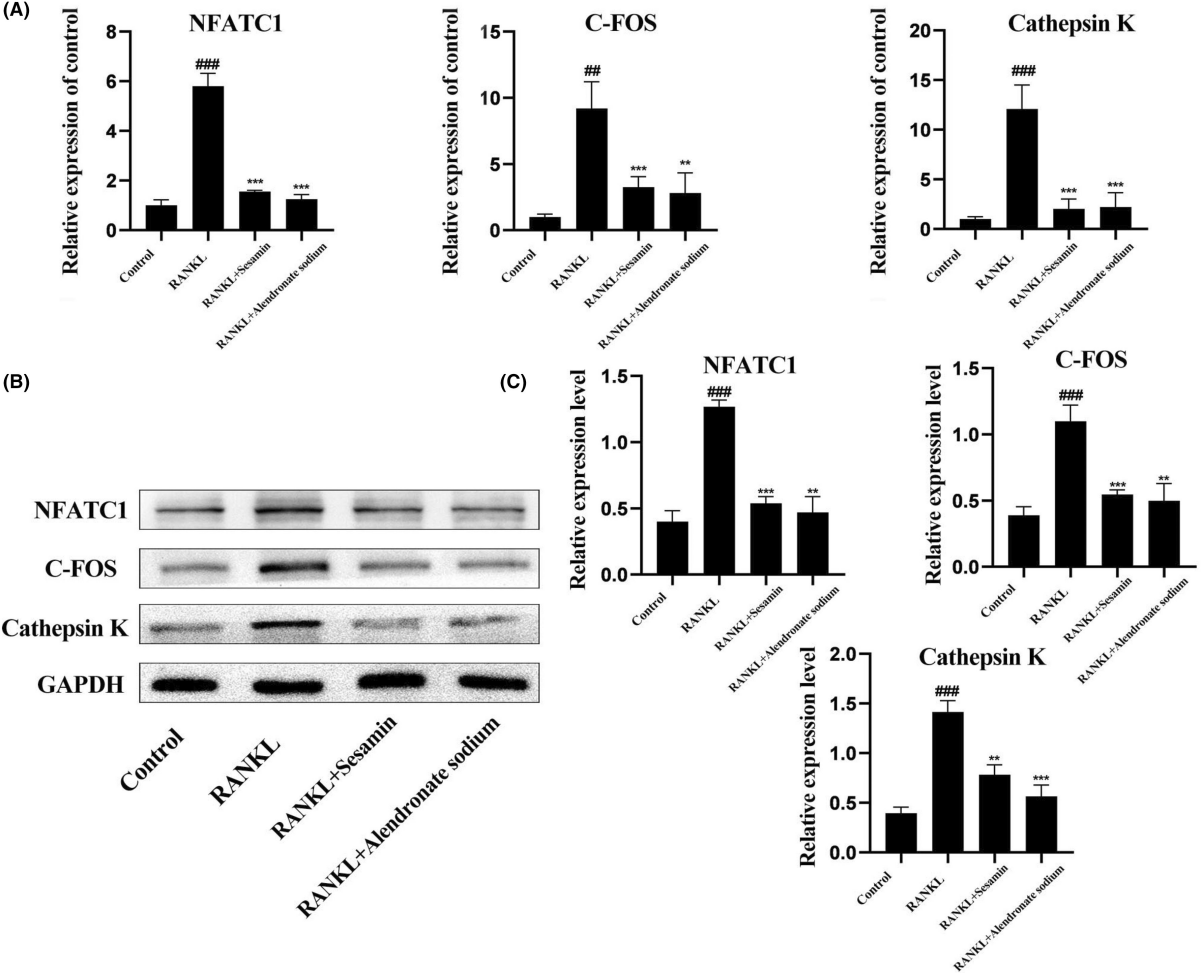
Sesamin and alendronate sodium inhibited RANKL‐induced osteoclast‐specific gene and protein expression.(A, B)The results of RT‐qPCR and western blot revealed that both sesamin and alendronate inhibited the expression of NFATC1, C‐FOS and Cathepsin K genes and protein relative to the RANKL‐treated group and were not statistically different from each other.(C) Quantification of western blot analysis. Data are presented as the mean ± SD (^##^ *p* < 0.01, ^###^ *p* < 0.001 vs. the control group and **p < 0.01, ***p < 0.001 vs. the RANKL alone treatment group).

Alendronate sodium, an official drug targeting osteoclasts, could inhibit osteoclastogenesis and prevent bone mineral‐related diseases. We further evaluated the suppression effect of alendronate sodium on RANKL‐induced osteoclast‐specific gene and protein expression, as a positive group, compared with sesamin treatment. RAW264.7 cells were treated with an induction medium containing RANKL and cultured with sesamin and alendronate. The results of RT‐qPCR showed that sesamin and alendronate inhibited the expression of NFATC1, C‐FOS and Cathepsin K genes relative to the RANKL‐treated group (Figure 4A). The western blot results demonstrated that sesamin and alendronate significantly decreased the protein expression levels of NFATC1, C‐FOS and Cathepsin K compared with the RANKL‐treated group (Figure 4B,C). Therefore, the results revealed that sesamin and alendronate could inhibit the expression of RANKL‐induced osteoclast‐specific gene and protein during osteoclast differentiation in vitro.

### 3.5 Sesamin suppresses ERK and NF‐κB signalling pathways during osteoclastogenesis

**FIGURE 5:**
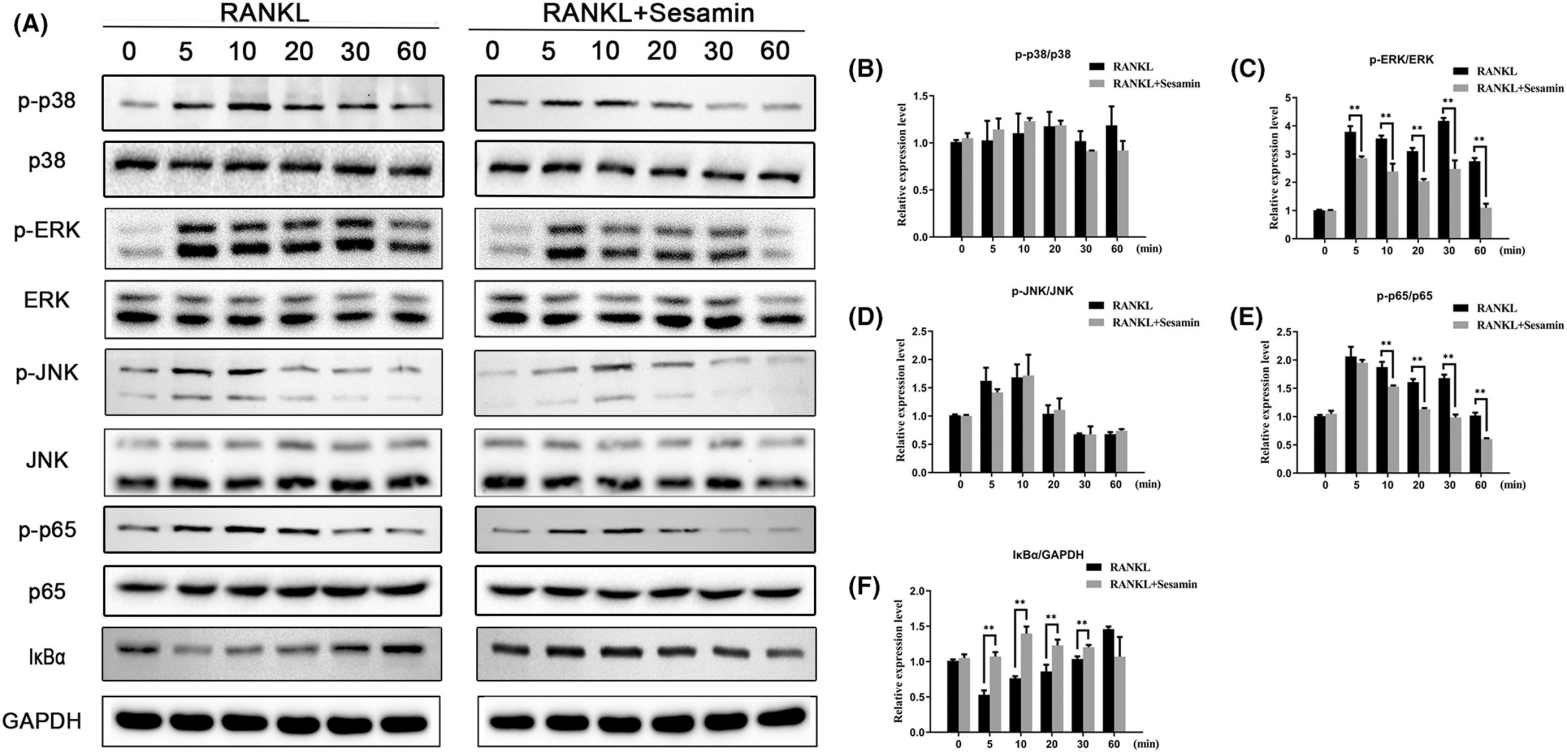
Sesamin suppressed RANKL‐induced ERK and NF‐κB signalling pathways. (A) RAW264.7 cells were pretreated with or without 5 μM sesamin for 4 h followed by RANKL (50 ng/mL) as indicated. Cell lysates were then analysed using Western blotting with antibodies against phosphor‐p38 and p38, phosphor‐ERK and ERK, phosphor‐JNK and JNK, phosphor‐p65 and p65 and IκBα. (B–F) Quantitative analysis was performed using ImageJ software, and the following values were calculated: p‐p38/p38, p‐ERK/ERK, p‐JNK/JNK, p‐p65/p65, IκBα/GAPDH. Data are presented as the mean ± SD (**p < 0.01, vs. control group).

**FIGURE 6:**
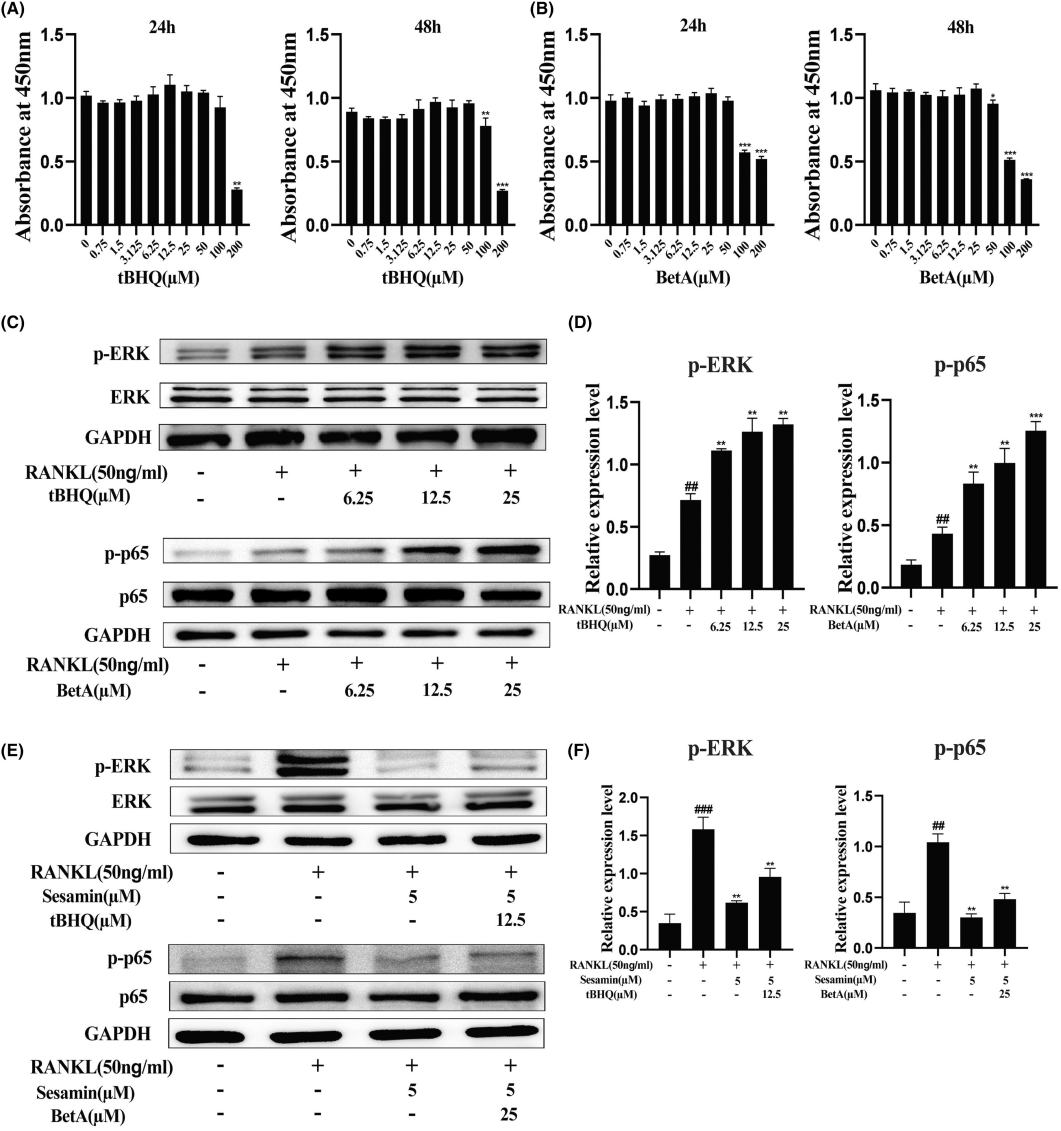
Sesamin antagonized the activation of ERK and NF‐κB pathway after tBHQ and BetA treatment. (A, B) The RAW264.7 cells were cultured with different concentrations of tert‐Butylhydroquinone (TBHQ) and Betulinic acid (BetA) for 24 or 48 h. Cell viability was measured using the CCK‐8 assay. (C) BetA and tBHQ significantly increased the protein expression levels of p‐p65 and p‐ERK compared with the RANKL alone treatment group. (D) Quantification of western blot analysis for the protein expression levels of p‐ERK and p‐p65. (E) BetA and tBHQ reversed the effects of sesamin in inhibiting phosphorylation of p65 and ERK, respectively. (F) Quantification of western blot analysis, and the following values were calculated: p‐ERK and p‐p65. Data are presented as the mean ± SD (##*p* < 0.01, ###*p* < 0.001 vs. the control group and **p < 0.01 and ***p < 0.001 vs. the RANKL alone treatment group).

The inhibitory effect of sesamin on the formation and function of osteoclasts indicated that sesamin had a potential role in the regulation of osteoclast formation. Therefore, we performed western blotting to measure the effect of sesamin on the expression of various signalling proteins to clarify the possible underlying mechanisms. By detecting the changes of related proteins, it was found that sesamin had no obvious effect on the activation of JNK and P38 of the MAPK subfamily, but was closely related to the activation of ERK signalling pathways (Figure 5A–D). In addition, to further explore the effect of sesamin on NF‐κB activation, we investigated the effect of sesamin on the degradation of IκBα and phosphorylation of p65 protein after RANKL stimulation on RAW264.7 cells at different time periods after pre‐treating with 5 μM sesamin for 4 h. The results demonstrated that sesamin had a significant inhibitory effect on RANKL‐induced IκBα degradation and p65 phosphorylation (Figure 5E,F). To further verify the role of the ERK and NF‐κB signalling pathways in the mechanisms of sesamin on osteoclast formation, tBHQ (an activator of ERK1/2) and BetA (a new activator of NF‐κB) were added to RAW264.7 cells.^22^, ^23^ Firstly, we examined the effect of tBHQ and BetA on RAW264.7 cells viability by the CCK‐8 assay. Our data revealed that the dose of tBHQ < 100 μM and < 50 μM had no cytotoxic effect on RAW264.7 cells after a treatment for 24 and 48 h, respectively (Figure 6A). The safe concentration of BetA on RAW264.7 cells were 0–50 μM and 0–25 μM for 24 and 48 h, respectively (Figure 6B). The western blot results demonstrated that tBHQ and BetA significantly increased the protein expression levels of p‐ERK and p‐p65 compared with the RANKL alone treatment group (Figure 6C,D). Notably, tBHQ and BetA reversed the effects of sesamin in inhibiting phosphorylation of ERK and p65, respectively (Figure 6E,F). Taken together, these above results indicated that sesamin suppressed the RANKL‐induced ERK and NF‐κB signalling pathways.

### 3.6 Molecular docking results

**FIGURE 7:**
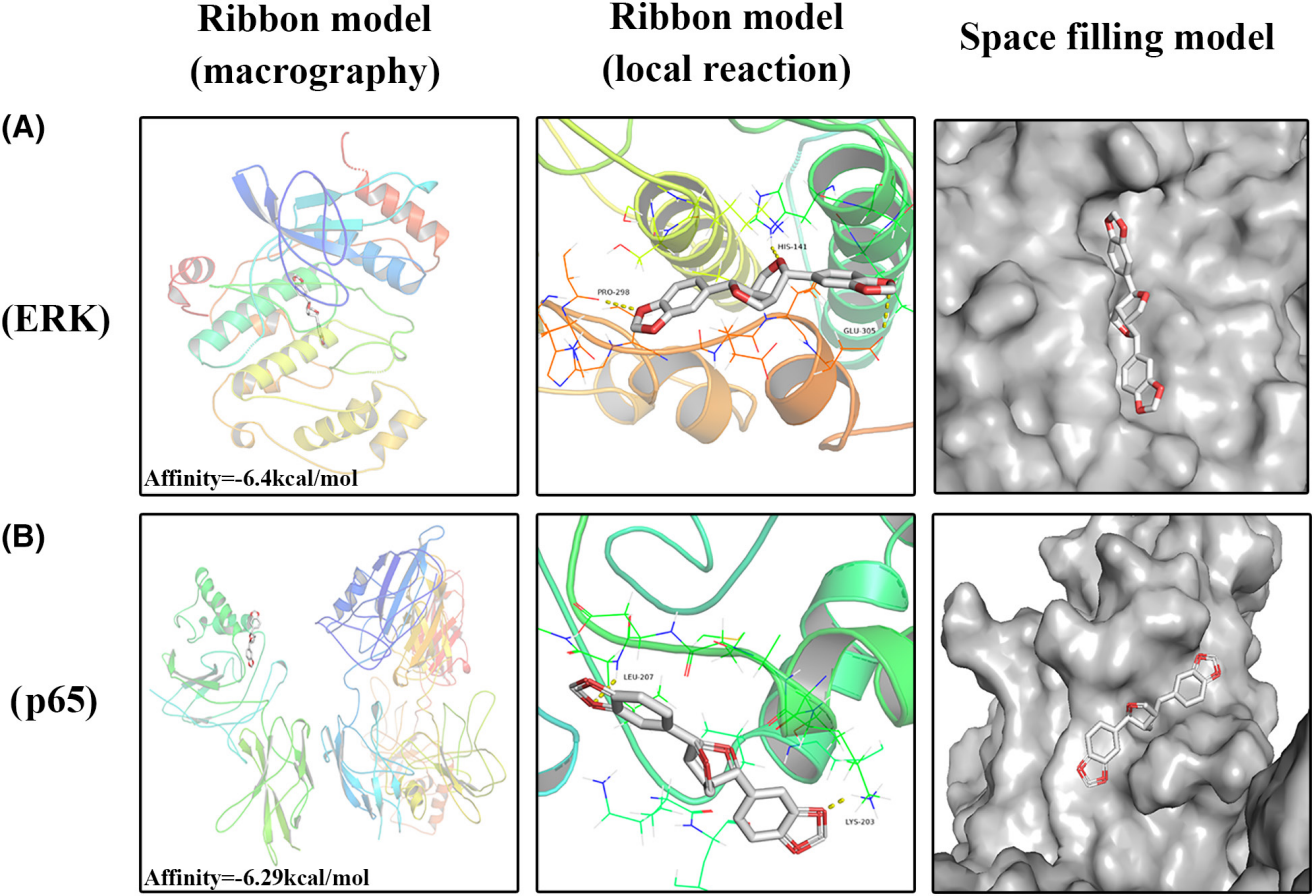
Sesamin was docked to ERK and p65 structures. (A, B) Macroscopic views and space‐filling models showed that sesamin was well embedded in the ERK and P65 protein binding pockets. (A) Protein residue interactions revealed the formation of hydrogen bonds between sesamin and ERK for PRO‐298, HIS‐141 and Glu‐305. (B) Hydrogen bonds of LEU‐207 and LYS‐203 were formed between sesamin and p65.

Western blotting results showed that sesamin was involved in regulating ERK and NF‐κB signalling pathways. To further analyse the binding affinity between sesamin and pockets of receptor protein, we performed a molecular docking analysis of sesamin with ERK and P65 proteins. Through space‐filling models and macroscopic views, we could observe that sesamin was well embedded in the ERK and P65 protein binding pockets (Figure 7A,B). In addition, local interaction views of protein residues were shown in the ribbon model. A number of important hydrogen bonds were formed between sesamin and ERK, which were associated with PRO‐298, HIS‐141 and Glu‐305 with an affinity of −6.4 kcal mol‐1 (Figure 7A). Similarly, as shown in Figure 7B, sesamin docked with the p65 structure and showed strong affinity at −6.29 kcal mol‐1 by forming hydrogen bonds with LEU‐207 and LYS‐203.

### 3.7 Sesamin protects LPS‐induced osteolysis in vivo

**FIGURE 8:**
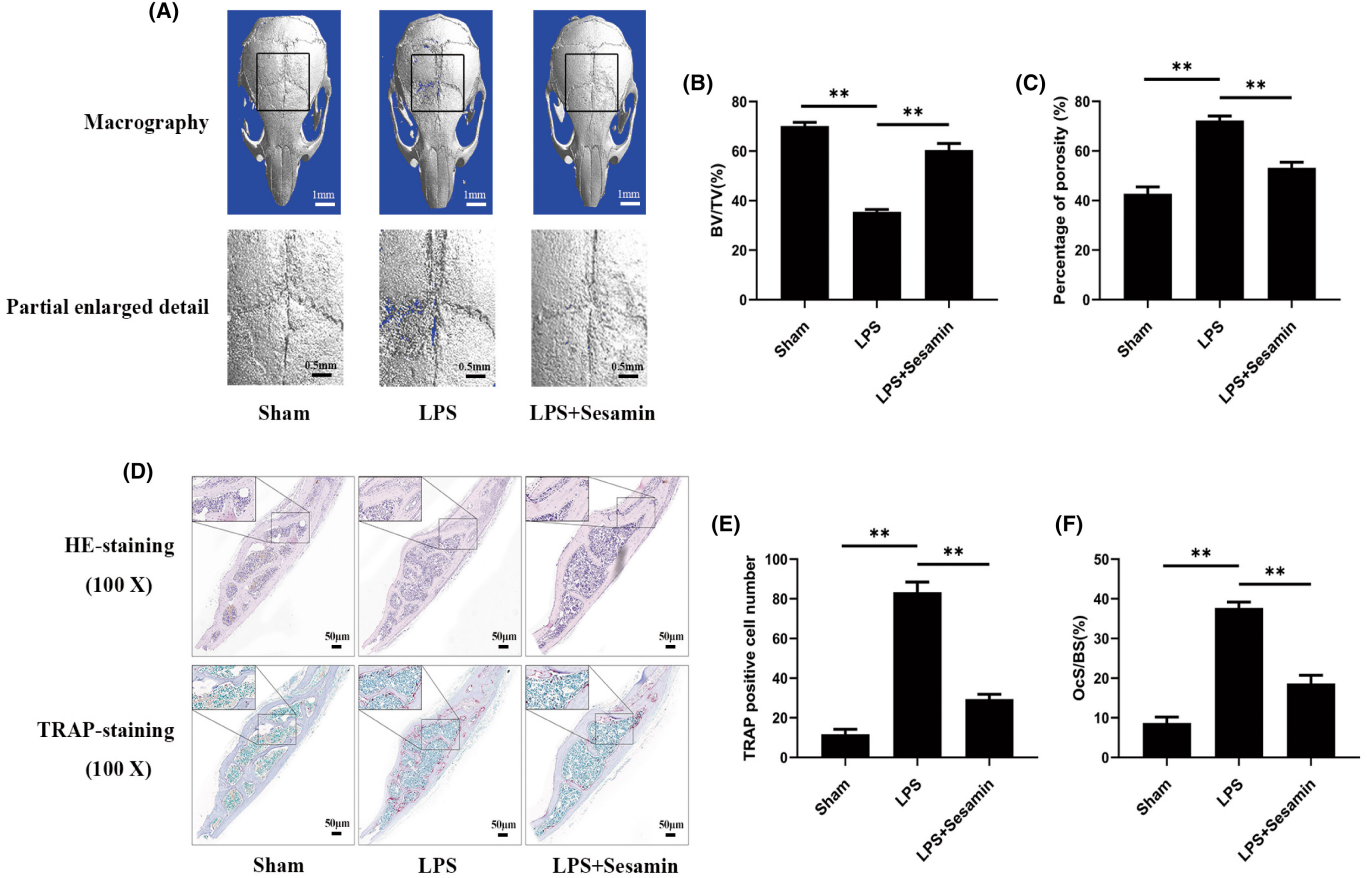
Sesamin protected LPS‐induced osteolysis in vivo. (A) Micro‐CT 3D reconstructed radiograph of harvested murine calvaria in each group. (B, C) The bone volume versus tissue volume (BV/TV) and percentage of porosity within regions of interest were measured. (D) Haematoxylin and eosin (HE) and tartrate‐resistant acid phosphatase (TRAP) staining were performed. (E,F) TRAP positive multinucleated osteoclasts and osteoclast surface versus bone surface (OcS/BS) were calculated. Data are presented as the mean ± SD (*p < 0.05 and **p < 0.01, vs. LPS group).

After observing the effect of sesamin on osteoclasts in vitro, we constructed a mouse model of the calvaria LPS‐induced osteolysis to study the effect of sesamin on the prevention of bone destruction in vivo. Micro‐CT scanning and 3D reconstruction of the calvaria showed that substantial bone erosion was observed in the LPS group compared to the control group (Figure 8A). Conversely, sesamin treatment effectively decreased the severity of osteolysis, which was further evidenced by the reduction in bone morphological indices including BV/TV and the percentage of porosity (Figure 8A–C).

**FIGURE 9:**
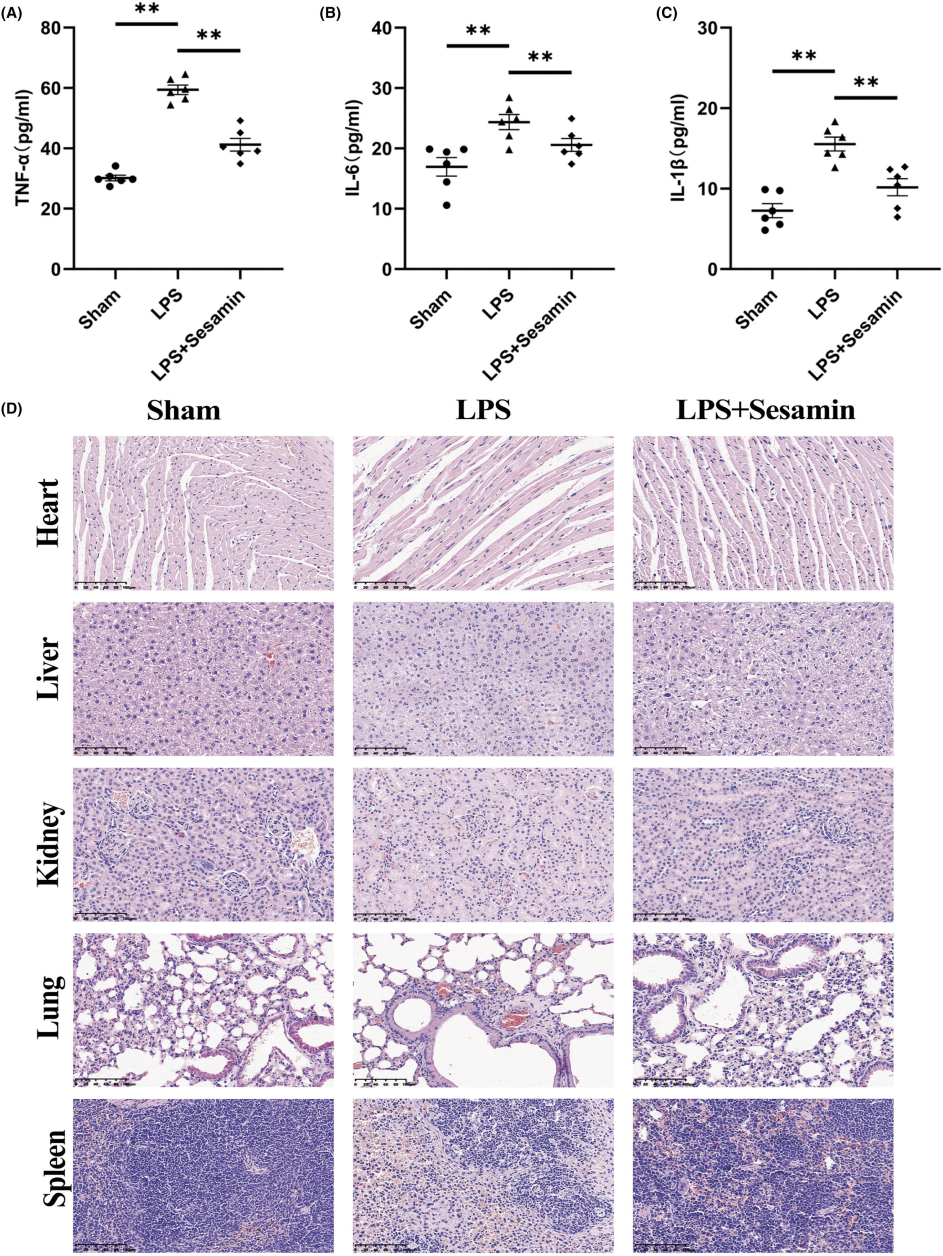
Sesamin reduced production of pro‐inflammatory cytokines in vivo and was non‐toxic to heart, liver, kidney, lung and spleen. (A–C) The levels of TNF‐α, IL‐6 and IL‐1β in mouse serum were assessed by ELISA. Data are presented as the mean ± SD (*p < 0.05 and **p < 0.01, vs. LPS group). (D) Haematoxylin and eosin staining was applied for the damage detection of sesamin to the main organs of mice, including heart, liver, kidney, lung and spleen.

HE and TRAP staining showed that an increase in the number of TRAP‐positive osteoclasts and a significant increase in the osteoclast surface versus bone surface (OcS/BS) in the LPS group (Figure 8D), resulted in significant bone erosion and trabecular microstructure damage. Compared with the LPS group, sesamin treatment group considerably protected the calvaria from bone erosion by reducing the number of osteoclasts and OcS/BS (Figure 8D–F). The quantitative analysis of sesamin on the reduction of bone erosion area and accumulation of osteoclasts showed similar results to those of micro‐CT findings. Moreover, ELISA kits were performed to detect concentrations of pro‐inflammatory cytokines including TNF‐α, IL‐6 and IL‐1β. The results exhibited that sesamin observably reduced the pro‐inflammatory cytokines production in mouse serum (Figure 9A–C). The damage of sesamin to the main organs of mice, including heart, liver, kidney, lung and spleen, was detected by HE staining, and the results are shown in Figure 9D. It could be seen that the heart, liver, kidney, lung and spleen tissue of mice were arranged tightly and regularly. Cells had a regular structure. These phenomena demonstrated that sesamin was non‐toxic to heart, liver, kidney, lung and spleen. Taken together, the in vivo results supported that sesamin could prevent LPS‐induced osteolysis by inhibiting osteoclast activity and pro‐inflammatory cytokines production.

## 4 DISCUSSION

Inhibition of pro‐inflammatory cytokines production and osteoclast differentiation is a promising strategy for the treatment of osteolytic diseases.^24^ However, there are limited drugs available to inhibit osteolysis, and the drugs currently in clinical use can cause numerous side effects, such as osteonecrosis of the jaw, oesophageal ulcers and increased risk of oesophageal tumours.^25^ Therefore, it is essential to find a drug that can inhibit the secretion of pro‐inflammation cytokines and osteoclastic bone resorption functions with fewer side effects to prevent osteolysis. Pharmacodynamic studies of plant monomers have become a hot topic of research in the treatment of osteolytic diseases.^26^, ^27^, ^28^ Here, we studied the effects of sesamin on osteoclast differentiation and pro‐inflammatory cytokines generation.

Firstly, in an in vitro experiment, to clarify whether sesamin has an inhibitory effect on osteoclasts, we performed CCK‐8 assay to determine the safe concentration range of the sesamin on BMMs. The appropriate concentration of sesamin in this range was selected to interfere with the RANKL‐induced osteoclast differentiation process, thus excluding the inhibition of osteoclast differentiation caused by cytotoxicity. The results showed that sesamin at concentrations less than 50 μM did not affect the activity of BMMs. Subsequently, we selected different concentrations of sesamin (0, 2.5, 5, 10 μM) to explore whether it interfered with osteoclast differentiation. Sesamin was found to inhibit osteoclast differentiation in a significant dose‐dependent manner, which was consistent with the report by Orawan et al.^29^

Mature osteoclasts have a lot of obvious pseudopodias and folded areas on the actin rings, which are critical structures where osteoclasts can attach to the surface of bone tissue to perform bone resorption functions.^30^ Our study revealed that sesamin significantly inhibited the formation of F‐actin rings in osteoclasts, and the fluorescence intensity of F‐actin rings decreased correspondingly with the increase of sesamin concentration. In addition, osteoclasts are the only cells that act as bone resorption functions, which secrete Cathepsin K to degrade bone.^31^ Our study found a significant reduction of bone resorption areas in sesamin‐treated bovine bone slices, indicating that sesamin could inhibit the osteoclastic bone resorption function. Taken together, the above results suggested that sesamin could inhibit the conversion of osteoclast precursors to mature osteoclasts and suppress osteoclast function.

Both osteoclast formation and bone resorption processes are regulated by specific genes, and upregulation of these genes can be served as a marker to verify the stage of osteoclast differentiation from the genetic level.^32^ Our results showed that sesamin treatment at concentrations of 5 and 10 μM significantly inhibited the expression of a number of osteoclast‐specific genes, including downstream transcription factor genes (NFATc1 and c‐fos),^33^ osteoclast bone resorption genes (TRAP and Cathepsin K),^34^, ^35^ maintenance of calcium homeostasis gene (CTR),^36^ and the precursor cell fusion genes (V‐ATPase d2, V‐ATPase a3 and DC‐STAMP).^37^, ^38^, ^39^

Previous studies have demonstrated the potential of sesamin to treat a variety of inflammatory diseases. For example, Chen et al. showed that sesamin protected against DSS‐induced colitis in mice by inhibiting NF‐κB and MAPK signalling pathways and Kang Li's research proves that sesamin can protect intervertebral disc from inflammation and extracellular matrix catabolism by affecting JNK pathway.^40^, ^41^ Sesamin has been studied in treating osteoporosis, inhibiting osteoclast differentiation and promoting osteoblast differentiation.^29^, ^42^ Previous studies have focused on degenerative diseases such as intervertebral disc degeneration and osteoporosis, but the osteolytic changes caused by LPS and the role of sesamin in osteolytic diseases have not been considered. We have proved that sesamin can mediate the occurrence and development of osteolytic diseases by affecting the classical NF‐κB and ERK pathways. Furthermore, Jeng et al. concluded that sesamin suppressed LPS‐induced IL‐6 production by inhibiting the p38 MAPK signalling pathway and NF‐κB activation.^43^ In the present study, we demonstrated that sesamin inhibited RANKL‐induced NF‐κB signalling by blocking the degradation of IκBα and phosphorylation of p65. In addition, Orawan et al. reported that sesamin stimulated osteoblast differentiation via p38 and ERK1/2 MAPK signalling pathways.^44^ Interestingly, we found that sesamin affected MAPKs signalling in a different manner between osteoblast and osteoclast differentiation. Sesamin were involved in inhibiting ERK activation without affecting JNK, p38 activation in the MAPK signalling cascade during osteoclastogenesis. This discrepancy may be due to the diversity of the cellular and animal models used in these researches and the limited exploration of molecular mechanisms.

LPS induces bone loss by stimulating release in pro‐inflammatory cytokines, which in turn promotes osteoclastogenesis and activates the effects of bone resorption.^45^, ^46^ Therefore, LPS is widely used to establish animal models of osteolysis.^47^, ^48^, ^49^ We established a mouse model of LPS‐induced osteolysis to investigate whether sesamin has the ability to inhibit pro‐inflammatory cytokines generation and osteoclastogenesis. Micro‐CT scanning showed a decrease in bone volume (BV/TV) and a significant increase in bone porosity in LPS‐treated group mice, which indicated the model of LPS‐induced osteolysis was successfully established. After sesamin treatment, the bone volume (BV/TV) of calvaria samples was more than that of LPS control group, and the bone porosity was significantly reduced. Consistent with the data shown by micro CT, haematoxylin and eosin and TRAP staining results revealed that sesamin effectively inhibited LPS‐induced osteolysis in vivo. Furthermore, in vivo expression of TNF‐α, IL‐6 and IL‐1β were significantly reduced by sesamin treatment relative to the LPS group, which suggested that sesamin might prevent osteolysis by suppressing inflammatory reaction.

Despite these encouraging in vitro and in vivo results, there are several limitations to our study. Firstly, analysis of upstream signalling proteins may be needed to make the evidence more adequate. Secondly, this study only investigated the levels of a limited number of signalling proteins, so further studies may be needed to elucidate other mechanisms of sesamin influencing on osteolysis. Finally, we did not further analyse the chemical reactivity and pharmacological properties of sesamin, which will be the focus of our follow‐up work.

**FIGURE 10:**
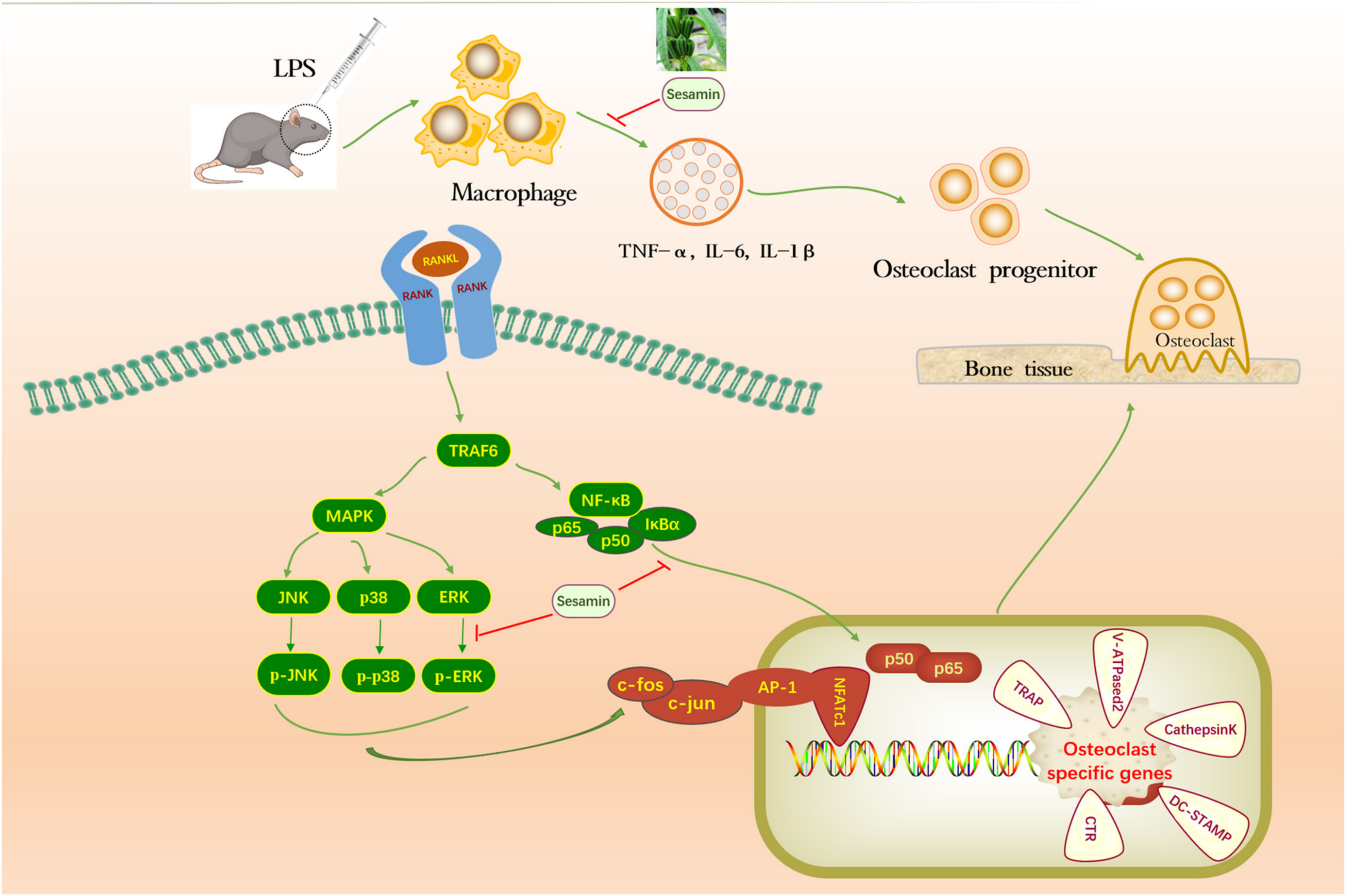
Schematic illustration of the inhibitory effect of sesamin on osteoclast formation and pro‐inflammatory cytokines secretion.

In conclusion, in vitro experiments revealed that sesamin could inhibit the differentiation and function of osteoclasts. In addition, in vivo experiments demonstrated that sesamin could reduce the production of pro‐inflammatory cytokines and inhibit the formation of osteoclasts, furthermore inhibiting LPS‐induced osteolysis. The intrinsic mechanism might be that sesamin inhibited osteoclast differentiation and specific gene expression by suppressing ERK and NF‐κB signalling pathways (Figure 10). Thus, sesamin could be a potential drug for the treatment of inflammatory and osteoclast‐related osteolytic diseases.

## AUTHOR CONTRIBUTIONS

**Xiaolong Yu:** Writing – original draft (equal). **Jiawei Hu:** Investigation (equal); visualization (equal). **Xinming Yang:** Visualization (equal). **Qiang Xu:** Resources (equal); software (equal). **Hangjun Chen:** Software (equal); validation (equal). **Ping Zhan:** Funding acquisition (equal); writing – review and editing (equal). **Bin Zhang:** Funding acquisition (supporting); validation (equal); visualization (equal).

## FUNDING INFORMATION

This work was supported by the National Natural Science Foundation (Grant No. 81860405: 82301001); Chinese Medicine Science and Technology Program of Jiangxi Province (Grant No. 2023B1217); Natural Science Foundation of Jiangxi Province (Grant No. 20232BAB206052: 20202BABL206037) and The First Affiliated Hospital of Nanchang University young talents research and Cultivation fund (Grant No. YFYPY202249).

## CONFLICT OF INTEREST STATEMENT

The authors declare that the research was conducted in the absence of any commercial or financial relationships that could be construed as a potential conflict of interest.

## Data Availability

The data that support the findings of this study are available from the corresponding author upon reasonable request.
